# Tobacco Use and Parental Monitoring—Observations from Three Diverse Island Nations—Cook Islands, Curaçao, and East Timor

**DOI:** 10.3390/ijerph17207360

**Published:** 2020-10-09

**Authors:** Masood Ali Shaikh, Zahra Zare, Kwok W. Ng, Karen L. Celedonia, Michael Lowery Wilson

**Affiliations:** 1 Injury Epidemiology and Prevention Research Group, Turku Brain Injury Centre, Turku University Hospital and University of Turku, FI-20014 Turku, Finland; masoodalishaikh@gmail.com (M.A.S.); zare.zahra59@yahoo.com (Z.Z.); karen.k.celedonia@utu.fi (K.L.C.); 2 School of Educational Science and Psychology, University of Eastern Finland, FI-80101 Joensuu, Finland; kwok.ng@uef.fi; 3 Department of Physical Education and Sport Sciences, University of Limerick, V94 T9PX Limerick, Ireland; 4 Heidelberg Institute of Global Health (HIGH), Heidelberg University, 69120 Heidelberg, Germany

**Keywords:** tobacco use, smoking consumption, parental monitoring, adolescent health, epidemiology

## Abstract

Tobacco use among adolescents is a global problem of public health importance. This study examined the profile of differences and similarities in adolescent tobacco use, and the role of parental monitoring activities among adolescents in three island nations of varying economic status: Cook Islands, Curaçao, and East Timor. Using nationally representative data we conducted regression modeling to determine the effect of four types of parental monitoring activities on tobacco use. Within a recall period of 30 days prior to being surveyed, 29.7% of students in East Timor, 21.6% in Cook Islands, and 13.1% in Curaçao reported having smoked cigarettes and/or used tobacco in other forms during 1 or more days during the preceding 30 days. Lower rates of parental monitoring as measured by four variables (parental understanding of problems and worries; knowing about how free time was being spent; going over things without approval; and checking to see if homework was done) were associated with higher percentages of adolescent tobacco use. Taken together the results underscore the need for increased parental involvement in programs which are designed to reduce tobacco use among adolescents.

## 1. Introduction

The consequences of tobacco use on health have been clearly communicated by many scientific authorities. The misuse and abuse of tobacco, alcohol, and other substances account for long-term consequences for both physical and mental health [1,2,3]. Approximately 6 million lives are lost worldwide due to tobacco-related diseases every year [4]. Tobacco use remains as a major public health concern, especially in low- and middle-income countries (LMICs). LMICs account for more than 80% of the world’s smokers and are where 5.81 million deaths occurred in 2013 as a result of smoking [5,6,7]. According to a World Health Organisation (WHO) global report, the prevalence of tobacco use has decreased faster in high-income countries relative to LMICs. In addition, the numbers of smokers in LMICs have steadily risen since 2000 [8]. Global surveys have demonstrated that approximately 90% of smokers start smoking tobacco when they are less than 18 years of age, with about 24 million of these early smokers being (about 7%) of adolescent age (13–15 years) between 2000–2017 [8,9]. Moreover, based on US national data, about 2.2% of middle- (12–13 years old) and 8% of high-school (14–18 years old) youth are current cigarette smokers [10].

Early experimentation and initiation of substance use is assumed to be a robust predictor of the problems of substance use later in life [11,12,13]. Smoking during early adolescence leads to dependence and continued use in adulthood because of the strongly addictive nature of tobacco [14,15]. Furthermore, the use of tobacco and other drugs before mid-adolescence leads to major health risks, psychiatric disorders, delinquency, antisocial behavior and later substance use behaviors [16,17,18].

According to a study of 68 LMIC countries, the mean prevalence of exposure to second-hand smoke in young adolescents was 55.9% and it remains a major public health problem. Second-hand smoking is significantly associated with an increased susceptibility for tobacco use in young adolescents [19,20]. Several factors might be related to this association. First, second-hand smoke exposure may be an indicator of social cues from peers, siblings, teachers or parents prompting smoking initiation. Second, neural pathways might be activated by nicotine exposure from second-hand smoke. Neural pathways lead to increase in the brain’s sensitivity to nicotine and gives rise to the willingness to smoke [20].

Several studies have presented empirical data on psychological, biological and sociodemographic factors as being highly associated with adolescent smoking behavior. Some of these are self-esteem, pubertal status, parental monitoring, peer smoking, gender, race/ethnicity, parent smoking, and the availability of cigarettes [21,22,23,24]. Parental monitoring is one family-related factor that has consistently been related to substance use behavior [25,26]. Existing literature suggests that family behavior is partly responsible for adolescent substance use and also this same process may be implicated in modifying the impacts of smoking related behaviors among adolescents [25,27,28].

Parental monitoring is perceived as a protective factor against substance use behavior [29]. In addition, studies confirm that even in diverse samples [30] with populations being at high-risk for smoking debut, less use was predicted due to increased parental monitoring [31,32,33]. In this regard, being closely monitored by a parent influences cognition and behaviors related to tobacco use among adolescents. According to the findings of these studies, willingness and initiation to smoking are the factors that decline as a result of increased parental monitoring [34,35].

Adolescence is a transitional period when children make the transition from primary to secondary school, and from pre- adolescence to adolescence. During early adolescence some of the most influential changes occur in the relationship between parents and their children. On the other hand, during this period, adolescents gravitate to their peers more and monitoring by parents has a tendency to decrease [36]. Consequently, adolescents are faced with increased risk for substance use [36,37,38]. However, despite the acknowledged effect of peers with regard to substance use behaviors, research has shown that this effect can be attenuated via parental monitoring even during the transition period.

In this study, the protective effects of parental monitoring were supported based on several reasons—a close relationship between parent and child decreases deviancy because the child’s identification is primarily with that of the parents and their value systems which define the attitudes and behaviors of the child. Therefore, children who use substances or even have a tendency to use and receive average levels of parental monitoring may be prevented from associating with deviant peers who do [39,40].

Regarding the role of strict parenting, findings from a study conducted to assess the relationship between parenting styles and adolescent substance use demonstrated that levels of substance use among adolescents having parents with authoritative (characterized by warmth and strictness) and indulgent (characterized by warmth but not strictness) parenting styles were less than among those having parents with authoritative and neglectful (characterized by neither warmth nor strictness) parenting styles. While, according to other studies, the highest level of tobacco and illegal substance use were associated with the neglectful and authoritative parenting style [41].

In order to reduce the effect of tobacco-related illnesses on both tobacco users and non-tobacco users, a 30% relative reduction in the global prevalence of tobacco smoking will be required as a global target. Therefore, in the present study, we compare 3 island nations which are from different world regions with diverse economic statuses and in terms of characteristics of adolescent tobacco use and parental monitoring.

According to a global youth tobacco survey, smoking in East Timor is a serious problem. East Timor is facing a rapidly growing epidemic of tobacco use especially among adolescents. Compared to other countries in the same Asia Pacific Region, the prevalence (51%) of current smokers among young people is significantly higher in East Timor. Findings of this study revealed that factors such as closed-peer smoking, parental smoking, having family discussion about harmful effects of smoking, having seen cigarette advertisements, smoking being allowed in areas such as schools, public places and home; and number of days people smoked in the home was significantly associated with current smoking [42]. On the other hand, unavailability of restrictive laws and regulations were clearly related to the continuously rising trend of smoking among adolescents in East Timor [43].

Also, according to global health survey, onset of tobacco use before reaching to adulthood among smokers is common in Cook Islands so that nearly one-quarter of those young smokers started smoking for the first time before the age of ten. Overall, 21.1% and 22.5% of students smoked cigarettes and any tobacco on one or more days during the past 30 days in Cook Islands, respectively. Most (84.4%) of these students first tried a cigarette before age 14 years. Moreover, it was reported that people smoked in the presence of 70.2% of these students on one or more days during the seven days preceding being surveyed and that parents or guardians (40.2%) used some form of tobacco [44].

The proportion of students in Curaçao who reported smoking was lower than compared to the average of Caribbean students and also they reported an early onset of substance use (before age 14) less than 13-to 15-year old students smoked. Overall, in Curaçao, the proportion of students who currently smoked cigarettes on at least one day during the 30 days before the survey was 9.0%. Among these students who ever smoked cigarettes, 60.5% of them tried a cigarette before the age of 14. Among Curaçao students, nevertheless, the proportion of smoking before the age of 14 reduced with age, which may be because of consumption of smoking among older students [45].

Despite the increase in available data, there still remains insufficient information to make robust estimates of tobacco use among adolescents, particularly in economically-diverse regions [8]. In addition, determining factors which modify smoking behavior during adolescence is of paramount importance to reduce longer term health consequences [35]. Therefore, the present study was conducted to assess and profile differences and similarities in adolescent tobacco use, and the role of parental monitoring activities among school-attending adolescents in three relatively unstudied island nations from three different WHO regions.

## 2. Methods

### 2.1. Sample

Inspired by previous research [26], data from the 2015 Cook Islands, 2015, Curaçao, and 2015 East Timor Global School-based Student Health Survey (GSHS) were used for secondary analysis. The GSHS were developed by the World Health Organization in collaboration with the United States Centers for Disease Control and Prevention (CDC), and conducted in collaboration with the national ministries of health, in each country. Detailed information on the data collection methods, questionnaire, procedures, and data are available at CDC website (http://www.cdc.gov/gshs/). Briefly, a two-stage cluster sampling design were used to facilitate collection of data representing students in years/classes 8 to 13 in Cook Islands, grade 1 to 6 in Curaçao, and class/grade 7 to 11 in East Timor.

At stage one, schools were selected with a probability proportional to their enrollment size. At stage two, classrooms within the selected schools were randomly selected and all students in selected classes were eligible to participate. The school response rate was 100%, student response rate was 65%, overall response rate was 65%, and 701 students participated in Cook Islands; school response rate was 96%, student response rate was 86%, overall response rate was 83%, and 2765 students participated in Curaçao; while school response rate was 100%, student response rate was 79%, overall response rate was 79%, and 3704 students participated in East Timor.

In all three countries, the class/grade levels were selected which are typically attended by students in the age range of 13 to 17 years. All respondent 13 years old or younger were recoded as 13 years old in Cook Islands; 12 years and younger as 12 years old; and 11 years or younger as 11 years old in East Timor, owing to their small number. While respondents 18 years or older were initially coded at 18 year olds. Participation in the survey was voluntary and all students were informed of the anonymous nature of the questionnaire. Answers were self-reported on a questionnaire with computer scannable answer sheet. With the exception of verifying heights and weights, no validation measures were used for the other responses in the survey, including the responses to items used for the present study.

### 2.2. Measurements

Current tobacco use as dependent variable was derived from two questions in the GSHS: “During the past 30 days, on how many days did you smoke cigarettes?” and “During the past 30 days, on how many days did you use any tobacco products other than cigarettes, such as . . .”; in each of the three countries different types of non-cigarette tobacco products were named. Response options for both questions were the same and ranged from “0 days”, “1 or 2 days”, “3 to 5 days”, “6 to 9 days”, “10 to 19 days”, “20 to 29 days”, or “All 30 days”. For the purpose of this analyses, participants were classified as current tobacco user, if they either reported having smoked a cigarette or used any tobacco product for 1 or 2 days or more times in the past 30 days.

Four parental monitoring questions were investigated independently as explanatory variables: “During the past 30 days, how often did your parents or guardians check to see if your homework was done?”, “During the past 30 days, how often did your parents or guardians understand your problems and worries?”, During the past 30 days, how often did your parents or guardians really know what you were doing with your free time?, and “During the past 30 days, how often did your parents or guardians go through your things without your approval?”. Response options for all four questions were the same and ranged from “Never”, “Rarely”, “Sometimes”, “Most of the time”, or “Always”. Responses of ‘most of the time’ and ‘always’ were combined for each question and coded as ‘yes’ while other responses were coded as ‘not having a monitoring parent’.

Additional questions on age when first tried a cigarette, having ever tried to stop smoking during the past twelve months, number of days other people smoked in the respondents’ presence during the last seven days, and parental tobacco use variables were also investigated.

### 2.3. Statistical Analysis

The prevalence and use of tobacco products, cigarette smoking, and other cigarette smoking related variables were examined first in terms of numbers and weighted percentages expressed as proportions. Differences between current tobacco use status and the various variables specified earlier were screened for statistical significance using Rao-Scott chi-square test, which is a design-adjusted version of Pearson’s chi-square test for categorical variables, and the design-adjusted version of *t*-test for continuous variable age. Statistical significance was considered at *p* < 0.05.

For each country, four survey binary logistic regression models were created to model the ability of each individual parental monitoring variable to predict its association with current tobacco use and not ’predict’ needs to be corrected. This was followed by inclusion of all four parental monitoring variables in separate binary logistic regression models for each country. The measures are reported as unadjusted odds ratios (OR), adjusted odds ratios(aOR), and associated 95% confidence intervals (CI). All analyses were carried out using Stata 16 (StataCorp, 2019).

## 3. Results

Within the recall period of 30 days prior to survey, 21.6% (95% CI: 18.3–25.3) students in Cook Islands, 13.1% (95% CI: 11.2–15.4) in Curaçao, and 29.7% (95% CI: 27.0–32.5) in East Timor reported having smoked cigarette and/or used tobacco in other forms one or more days. In Cook Islands, age and sex was missing for 3 and 5 records, respectively; while for 2 records both were missing. In Curaçao, age and sex was missing for 10 and 27 records; while for 3 records both were missing. And for East Timor, age and sex was missing for 74 and 202 records, respectively; while for 57 records both were missing.

Table 1 presents the descriptive statistics of tobacco use, smoking use and related questions in school attending adolescents in the Cook Islands, Curaçao, and East Timor. The proportion (percentage) of current cigarette smokers was 20.0%, and current tobacco users was 15.3% in Cook Islands; 9.0% and 7.7%, respectively for current cigarette smokers and tobacco users in Curaçao; and 22.8% for current cigarette smokers and 18.7% for current tobacco users in East Timor. Regarding people having smoked in the presence of respondents during the past seven days, for one or more days, 68.0% replied affirmatively in Cook Islands, 58.3% in Curaçao, and 79.7% in East Timor.

Table 2 presents the descriptive statistics and bivariate associations by current tobacco use status (smoked cigarette and/or used tobacco) in school attending adolescents in the three countries. Among tobacco users, 54.4% were males in Cook Islands, 57.7% in Curaçao, and 73.0% in East Timor. Statistically significant associations were found between gender and current tobacco use in Curaçao and East Timor only. Age and current tobacco use was also statistically significant in Curaçao and East Timor, with older students more likely to report tobacco use in any form. Albeit, this association was not statistically significant in Cook Islands.

Regarding parental monitoring variables; statistically significant bivariate associations were found between understanding of problems and worries by the parents in Curaçao, parents really knowing what was being done with free time in Cook Islands and Curaçao, and parents checking to see if homework was done in Curaçao and East Timor; with lower percentages of such monitoring activities in current tobacco users. For parents who went over respondent’s things without their approval, lower percentages were reported in all three countries by the current tobacco users. However statistically significant association was found in Curaçao and East Timor, but not in Cook Islands.

Table 3 presents the results of simple and multiple logistic regression modeling analysis of current tobacco use and parental monitoring activities among school attending adolescents in three countries. Odds ratio (OR) based on simple logistic regression models in each country were statistically significant, for the associations between current tobacco use and understanding of problems and worries by the parents in Curaçao, parents really knowing what was being done with free time in Cook Islands and Curaçao, and parents checking to see if homework was done in Curaçao and East Timor; with ORs lower than one that is, parental monitoring behaviors bestowed protection from tobacco use. Association between current tobacco use and parental activity of going over respondents’ things without their approval was statistically significant and above one in Curaçao and East Timor, with lower percentages of such monitoring activities in current tobacco users.

When adjusting for all covariates in the multiple logistic regression model, association of current tobacco use and understanding of problems and worries by the parents was not statistically significant for any country. Other parental monitoring activities, where statistically significant, bestowed protection from current tobacco use. However, with the exception of parents going over things without respondent’s approval; which was associated with higher adjusted odds ratios (aOR) for current tobacco use. The goodness-of-fit test revealed that multivariate logistic regression models with parental monitoring covariates in all three countries were good models for tobacco use.

## 4. Discussion

The aim of this study was to determine the prevalence of adolescent tobacco use in three economically-diverse island nations and explore whether parental monitoring activities were associated with adolescent tobacco use among school-attending adolescents in these countries. This study revealed that within the recall period, 29.7% students in East Timor reported having smoked cigarette and/or used tobacco in other forms one or more days, which is a higher prevalence compared to Cook Islands and Curaçao, with 21.6% and 13.1%, respectively. The highest prevalence of tobacco smoking was previously reported to be in the South-East Asian Region within East Timor among young people (13–15 years old), and also that the prevalence of tobacco use was reported higher in LMICs (like East Timor) compared to high-income (Curaçao) and upper middle-income (Cook Islands) countries, which was consistent with our findings [8].

In this study, East Timor accounted for 22.8% for current cigarette smokers and 18.7% for current tobacco users, with Cook Islands and Curaçao having lower proportions, respectively. According to the Global Youth Tobacco Survey (GYTS), East Timor had the highest median current tobacco smoking prevalence among students aged 13–15 years from 2012 to 2015 across 61 countries, with the prevalence of tobacco use and cigarette smoking being lowest in the European region [46]. In addition, according to the Global Youth Tobacco Survey on 45 countries (six WHO regions) in 2013 and 2014, East Timor had the highest median level of overall current cigarette smoking prevalence among students aged 13–15 years [47].

Regarding people having smoked in the presence of respondents during the seven days preceding being asked, for one or more days, 68.0% replied affirmatively in Cook Islands, 58.3% in Curaçao, and 79.7% in East Timor in the present study. As reported elsewhere, the highest prevalence of exposure to secondhand smoke globally was 63.5% in the western Pacific region [19].

Males were over-represented as being current tobacco users in each of the studied countries. This was generally consistent with prior research examining tobacco use among adolescents in these three settings [46,47]. However it contradicts findings in other world regions where females are generally perceived to have a higher predisposition toward smoking initiation and continuance [48]. It has also been suggested that girls might be more susceptible to the effects of prenatal exposure to smoking parents [49]. It is not entirely clear why smoking behavior differs in this regard by gender. It is fully possible that cultural norms around what might be expected of boys vs girls may attenuate smoking behavior among girls. There may also be social factors in risk perception by gender which might help to explain this variation [50]. Not only was proportion of current tobacco users among boys higher in all three countries, statistically significant associations were found between male gender and current tobacco use in Curaçao and East Timor.

East-Timor had the highest values in tobacco smoking generally. There may be some cultural undertones to this finding. A tradition of indicating appreciation by giving out free cigarettes in some communities in East-Timor may be one of the reasons why underage children may be more exposed to smoking initiation. Socioeconomic status is another potential reason. Selling loose cigarettes by under age children in order to earn money may decrease any existing social barriers around smoking initiation since the these children have access to cigarettes in some quantity. A third reason may result from the absence of legislation restricting cigarette sales and consumption by minors. Cigarettes are accessible in small shops and are affordable in East-Timor [42].

The first age for starting to smoke was 11 years old or less for East Timor, 12 years old or less for Curaçao, and 13 years old or less for Cook Islands. Different studies reported the first age when students tried to initiate smoking. In a study, conducted on islands of the pacific region, half of cigarette users tried smoking before age 14 for the first time [51]. According to the Global Youth Tobacco Survey, the first age for starting to smoke was before the age of 11 years among more than half of smokers in more than half of the countries that contributed to the GYTS [46].

With respect to parental monitoring activities, lower percentages of parental monitoring such as understanding of problems and worries, free time activities, checking homework, and went over things without approval was significantly associated with higher percentages of current tobacco use.

These results are congruent with previous research showing that parental monitoring having an influence on smoking-related cognition such as intentions and willingness - with decreased parental monitoring there was increased smoking initiation [34,35,52,53] The buffering effect of parental monitoring has been demonstrated by other studies, and it has been shown that the associations between tobacco use and peers with deviant behavior can be interrupted by parental monitoring during the transition from childhood to adolescence [32,39,54]. Consistent with previous research using the global-school-based student health survey (GSHS), which has showed parents’ lack of awareness of free time activities as a significant predictor of smoking, while parents who really knew what their children were doing during their free time was associated with less tobacco use [55].

In addition, according to other research, parental monitoring was targeted as a predictor for early onset of substance use among adolescents. Since youth spend more time with peers than parents during early adolescence, they may be exposed to peers who engage in substance use. Although adolescents are considerably influential in each others’ lives during adolescence, early initiation of substance use can be prevented by the protective role of parents such as monitoring children’s free time activities, who they are with and where they go [36,37,38]. In a longitudinal study, the protective role of parental monitoring against substance use was assessed in the 5th grade, and the results confirmed that using substances by youth in 9th grade was related to parental monitoring activities [56]. The study results presented here confirm other studies with regard to decreased use of substances resulted from increased monitoring activities across diverse population samples [30,31,32,33].

Some of the myriad activities that aid in reducing smoking and tobacco use behaviors among minors include: Encouraging stricter parenting behaviors via national prevention and educational programs; implementing national mass media campaigns and prevention programs; setting stricter policies at schools and stricter societal approaches toward adolescent tobacco use; changing socio-cultural norms regarding adolescent tobacco use; implementing bans on selling tobacco to minors; installing policies aimed at increasing physical activity and healthy eating among school children, and promoting positive changes in the way young people spend their leisure time have been widely recommended and are reiterated here [30,31,32,33].

Our findings should be considered within the limitations of the study. Due to the cross-sectional nature of the data, causal inference cannot be applied to the findings. Additionally, given that the survey data were self-reported, response bias may be present. Depending on the cultural acceptability of tobacco use in each of the respective countries, use may have either been under- or over-reported by youth. Response rates–and therefore, sample sizes–were not comparable across the three countries, which could have affected the analyses. Limitations aside, the present study provides useful data on adolescent tobacco use and parental monitoring in these three island nations, and adds to the growing body of literature on adolescent tobacco use epidemiology in LMICs.

## 5. Conclusions

The findings have implications for tobacco use prevention efforts in these countries and surrounding regions, suggesting that targeted public health initiatives designed to increase parental involvement in adolescents’ lives may help delay or entirely prevent the onset of tobacco use. More research needs to be done, however, to thoroughly understand the connection between tobacco use and parental monitoring in these countries.

## Figures and Tables

**Table 1:** Descriptive statistics of tobacco use and cigarette smoking in school attending adolescents.

	Cook Islands (*n* = 701)	Curaçao (*n* = 2765)	Timor-Leste (*n* = 3704)
**Current cigarette smoker**			
Yes	144 (20.0%)	229 (9.0%)	739 (22.8%)
No	546 (80.0%)	2500 (91.0%)	2805 (77.2%)
Missing	11	36	160
**Age first tried a cigarette (years)**			
Never smoked cigarette	349 (52.2%)	1848 (67.5%)	1897 (66.7%)
7 or younger	66 (9.2%)	85 (3.4%)	120 (4.1%)
8 or 9	64 (9.7%)	81 (2.9%)	67 (2.3%)
10 or 11	50 (7.2%)	134 (5.4%)	93 (3.5%)
12 or 13	61 (8.9%)	208 (8.0%)	99 (3.6%)
14 or 15	50 (7.3%)	182 (7.3%)	194 (7.3%)
16 or 17	31 (4.2%)	108 (4.5%)	205 (8.5%)
18 or older	9 (1.3%)	28 (1.0%)	90 (4.0%)
Missing	21	91	939
**Tried to quit cigarette smoking**			
Never smoked	423 (62.9%)	2068 (76.3%)	2213 (72.5%)
Didn’t smoke in past 12 months	119 (17.4%)	359 (14.2%)	164 (5.6%)
Yes	108 (15.1%)	149 (6.1%)	434 (16.5%)
No	31 (4.6%)	84 (3.4%)	160 (5.4%)
Missing	20	105	733
**Others smoked cigarette in my presence**			
0 days	220 (32.0%)	1160 (41.7%)	798 (20.3%)
1 or 2 days	157 (23.1%)	839 (31.0%)	1269 (34.3%)
3 or 4 days	71 (10.1%)	272 (10.2%)	326 (8.9%)
5 or 6 days	42 (5.7%)	86 (3.3%)	202 (5.4%)
All 7 days	206 (29.1%)	372 (13.8%)	1061 (31.1%)
Missing	5	36	48
**Current tobacco user in other forms**			
Yes	112 (15.3%)	203 (7.7%)	690 (18.7%)
No	586 (84.7%)	2525 (92.3%)	2963 (81.3%)
Missing	3	37	51

All frequencies are unweighted, while percentages are weighted.

**Table 2:** Descriptive statistics and bivariate associations by current tobacco use (cigarette and/or other forms) status in school attending adolescents.

	Cook Islands				Curaçao				East Timor			
	(*n* = 701 *)				(*n* = 2765 *)				(*n* = 3704 *)			
	Current Tobacco User											
	Yes		No		Yes		No		Yes		No	
	*n*	%	*n*	%	*n*	%	*n*	%	*n*	%	*n*	%
**Age (years)**												
11 years or less									46	1.2	79	1.9
12 years or less				13	0.4	245	6.7		29	0.7	121	2.4
13 years or less	8	1.1	55	8.3	26	0.7	297	8.7	69	1.5	250	4.9
14	26	4.1	128	18.9	63	2.2	401	14.1	123	2.8	440	9.8
15	37	5.1	110	15.9	65	2.4	400	15.6	136	3.5	450	11.9
16	39	4.7	127	17.8	51	2.3	300	12.3	185	5.5	464	13.5
17	34	4.8	86	12.6	49	2.1	306	12.6	229	7.4	492	16.2
18 years or more	12	1.7	34	5.0	77	3.0	442	16.9	199	6.9	295	9.9
Missing	1	–	2	–	1	–	8	–	30	–	44	–
Mean	15.6		15.3		15.8		15.4		16.0		15.6	
SD	1.4		1.4		1.7		1.9		1.8		1.8	
*p* value	0.080				0.001				0.002			
**Sex**												
Male	85	54.4	255	46.9	185	57.7	1031	47.4	665	73.0	951	41.5
Female	71	45.6	283	53.1	154	42.3	1347	52.6	310	27.0	1558	58.5
Missing	1	–	4	–	6	–	21	–	71	–	126	–
*p* value	0.1012				0.0024				<0.0001			
**Parents/guardians use tobacco**												
Neither	52	33.6	267	49.6	210	62.3	1861	78.3	384	39.7	1171	45.7
Father/Male guardian	34	21.9	83	15.1	54	17.6	286	11.8	208	18.8	385	14.8
Mother/Female guardian	20	13.1	60	11.8	32	8.5	112	4.8	70	6.2	57	2.1
Both	27	18.1	76	13.4	22	6.3	64	2.8	157	15.4	301	11.9
I do not know	23	13.3	55	10.1	18	5.3	56	2.3	193	19.9	682	25.5
Missing	1	–	1	–	9	–	20	–	34	–	39	–
**Parental monitoring (understanding)**												
Yes	41	27.2	156	29.0	128	40.7	1251	54.9	130	12.9	274	11.4
No	116	72.8	386	71.0	184	59.3	1018	45.1	871	87.1	2335	88.6
Missing value	0	–	0	–	33	–	130	–	45	–	26
*p* Value	0.7064				0.0003				0.4064			
**Parental monitoring (free time activities)**												
Yes	47	30.8	229	42.1	148	46.2	1525	67.2	255	25.3	546	22.7
No	108	69.2	313	57.9	164	53.8	733	32.8	736	74.7	2025	77.3
Missing value	2	–	0	–	33	–	141	–	55	–	64
*p* Value	0.0225				<0.0001				0.3222			
**Parental monitoring (went over things without permission)**												
Yes	24	15.5	76	14.8	46	14.8	218	9.8	96	9.9	155	6.6
No	133	84.5	466	85.2	266	85.2	2046	90.2	909	90.1	2428	93.4
Missing value	0	–	0	–	33	–	135	–	41	–	52	–
*p* Value	0.8177				0.0377				0.0127			
**Parental monitoring (checking homework)**												
Yes	40	25.6	174	32.9	79	24.1	813	34.9	251	25.7	766	29.7
No	116	74.4	368	67.1	229	75.9	1467	65.1	751	74.3	1841	70.3
Missing value	1	–	0	–	37	–	119	–	44	–	28	–
*p* Value	0.0868				0.0021				0.0221			

* For 2 records in Cook Islands, 21 records in Curaçao, and 23 records in East Timor, information on current tobacco use (defined as current cigarette smoker and/or current tobacco user) was missing. All frequencies are unweighted, while percentages are weighted.

**Table 3:** Multivariate analysis of current tobacco use and parental monitoring activities among school attending adolescents.

	Cook Islands	Curaçao	Timor-Leste
	OR	OR	OR
	OR (95%CI)	OR (95%CI)	OR (95%CI)
	*p* value	*p* value	*p* value
	aOR	aOR	aOR
	aOR (95%CI)	aOR (95%CI)	aOR (95%CI)
	*p* value	*p* value	*p* value
**Parental understanding**			
Yes	0.92	0.56	1.15
	0.58–1.45	0.41–0.77	0.81–1.64
	0.706	<0.0001	0.407
No	1.14	0.81	1.05
	0.55–2.37	0.58–1.13	0.73–1.53
	0.630	0.210	0.769
	1	1	1
**Parental monitoring (free time activities)**
Yes	0.61	0.42	1.15
	0.40–0.93	0.31–0.56	0.86–1.54
	0.23	<0.0001	0.322
No	0.63	0.48	1.17
	0.34–1.16	0.35–0.65	0.87–1.56
	0.046	<0.0001	0.277
	1	1	1
**Parental monitoring (went over things without permission)**
Yes	1.05	1.60	1.56
	0.67–1.66	1.02–2.49	1.11–2.19
	0.818	0.039	0.013
No	1.11	1.69	1.54
	0.63–1.98	1.04–2.74	1.19–2.00
	0.617	0.035	0.002
	1	1	1
**Parental monitoring (checking homework)**
Yes	0.70	0.59	0.82
	0.47–1.06	0.42–0.82	0.69–0.97
	0.088	0.002	0.022
No	0.76	0.71	0.76
	0.43–1.36	0.47–1.06	0.62–0.91
	0.213	0.097	0.006
	1	1	1

OR = Odds Ratio; aOR = Adjusted Odds Ratio.
